# A bacterial riboswitch class senses xanthine and uric acid to regulate genes associated with purine oxidation

**DOI:** 10.1261/rna.075218.120

**Published:** 2020-08

**Authors:** Diane Yu, Ronald R. Breaker

**Affiliations:** 1 Department of Molecular, Cellular and Developmental Biology, Yale University, New Haven, Connecticut 06520-8103, USA; 2 Department of Molecular Biophysics and Biochemistry, Yale University, New Haven, Connecticut 06520-8103, USA; 3 Howard Hughes Medical Institute, Yale University, New Haven, Connecticut 06520-8103, USA

**Keywords:** aptamer, dioxygenase, gene regulation, hypoxanthine, noncoding RNA, urate

## Abstract

Dozens of candidate orphan riboswitch classes have been discovered previously by using comparative sequence analysis algorithms to search bacterial genomic sequence databases. Each orphan is classified by the presence of distinct conserved nucleotide sequences and secondary structure features, and by its association with particular types of genes. One previously reported orphan riboswitch candidate is the “*NMT1* motif,” which forms a hairpin structure with an internal bulge that includes numerous highly conserved nucleotides. This motif associates with genes annotated to encode various dioxygenase enzymes, transporters, or proteins that have roles associated with thiamin or histidine metabolism. Biochemical evaluation of numerous ligand candidates revealed that *NMT1* motif RNA constructs most tightly bind 8-azaxanthine, xanthine, and uric acid, whereas most other closely related compounds are strongly rejected. Genetic assays revealed that *NMT1* motif RNAs function to turn off gene expression upon ligand binding, likely by regulating translation initiation. These results suggest that *NMT1* motif RNAs function as aptamer domains for a riboswitch class that specifically responds to high concentrations of oxidized purines. Members of this “xanthine riboswitch” class appear to regulate genes predominantly related to purine transport and oxidation, thus avoiding the effects of overproduction of these common purine derivatives.

## INTRODUCTION

Riboswitches that respond to metabolites or elemental ions are routinely used by many bacterial species to control the expression of genes coding for proteins that regulate metabolic or elemental ion homeostasis processes, or that make physiological changes in response to signaling molecules (Breaker 2011, 2012, 2018; Serganov and Nudler 2013; Sherwood and Henkin 2016; Lotz and Suess 2018). Over 45 different riboswitch classes have been experimentally validated, each using one or more characteristic aptamer domains to selectively bind a variety of fundamental metabolites or metal ions and control the expression of genes relevant to these ligands (McCown et al. 2017).

In addition, many classes of “orphan” riboswitch candidates (Barrick et al. 2004; Meyer et al. 2011; Weinberg et al. 2017a; Greenlee et al. 2018) have been discovered by using computational strategies to identify unusually well-conserved and structured RNAs located in the noncoding regions of some bacterial genomes. Both the number of orphan riboswitch candidates and the apparent diversity of these RNAs strongly suggest that far more riboswitch classes remain to be experimentally validated (Ames and Breaker 2010; McCown et al. 2017). If true, then the types of ligands sensed by riboswitch aptamers are likely to be far greater than is presently known. The discovery and validation of additional riboswitch classes and their associated regulatory networks will help reveal the functions of proteins whose activities were unknown or previously mischaracterized (e.g., see Baker et al. 2012; Nelson et al. 2017).

The *NMT1* motif is an orphan riboswitch candidate that was previously identified through comparative sequence analysis as a structured, noncoding RNA of unknown function (Weinberg et al. 2017a). Representatives of this RNA motif are usually found immediately upstream of the open reading frames (ORFs) of variously annotated genes wherein the precise functions of their protein products remain uncharacterized. One class of genes commonly associated with the *NMT1* motif codes for proteins commonly annotated as NMT1/THI5-like. These annotations imply that the genes share similarity to the *NMT1* gene of fungi, which codes for an enzyme involved in synthesis of the pyrimidine moiety of thiamin (McColl et al. 2003; Bale et al. 2010). There are already two validated riboswitch classes related to thiamin metabolism: one that senses the coenzyme form, thiamin pyrophosphate (TPP) (Mironov et al. 2002; Winkler et al. 2002) and another that senses the TPP precursor 4-amino-5-(hydroxymethyl)-2-methylpyrimidine pyrophosphate (HMP-PP) (Atilho et al. 2019). These observations were used to formulate our initial hypothesis that *NMT1* motif RNAs function as metabolite-binding riboswitches, perhaps for a ligand related to TPP metabolism.

However, further bioinformatic analysis of the *NMT1*/*THI5*-like annotated genes associated with the RNA motif revealed that these genes were distinct from the *NMT1* genes relevant to thiamin metabolism. Moreover, the various other genes associated with *NMT1* motifs were similar to those encoding non-heme iron (II) dependent dioxygenases, alpha-ketoglutarate dependent dioxygenases, nucleoside transporters, and adenosine and guanine deaminases. These bioinformatic analyses thus implicated the genes associated with *NMT1* motifs in purine degradation, rather than thiamin metabolism.

There are also various known riboswitch classes that sense purines and their biosynthetic intermediates, including guanine (Mandal et al. 2003), adenine (Mandal and Breaker 2004), ZTP (Kim et al. 2015), and phosphoribosyl pyrophosphate (PRPP) (Sherlock et al. 2018a). These riboswitch classes commonly regulate genes related to the production of purine nucleobases or purine transport. In addition, rare riboswitches for 2′-deoxyguanosine-5′-monophosphate (dGMP) regulate ribonucleotide reductase genes to control biosynthesis of purine DNA monomers (Kim et al. 2007; Weinberg et al. 2017b), and a riboswitch class also exists that senses 5′-diphosphorylated adenosine nucleotides ADP and dADP to regulate NUDIX hydrolases (Sherlock et al. 2019). Finally, several riboswitch classes exist that sense signaling molecules derived from purine nucleotides, including c-di-GMP (Sudarsan et al. 2008; Lee et al. 2010), c-di-AMP (Nelson et al. 2013), c-AMP-GMP (Kellenberger et al. 2015; Nelson et al. 2015a), and ppGpp (Sherlock et al. 2018b). All these experimentally validated riboswitch classes provide abundant precedence for aptamers that sense purines and their metabolites, demonstrating that the bacterial domain of life extensively exploits RNA to monitor the concentrations of various purines and their natural derivatives. Previously, however, there were no known riboswitch classes that predominantly regulate enzymes involved in the degradation of purine nucleobases.

In the current study, we demonstrate that *NMT1* motif RNAs selectively recognize oxidized purine nucleobases. Structure activity relationship (SAR) data reveal that natural ligands xanthine and uric acid are bound most tightly. In addition, genetic analyses demonstrate that representatives of these RNAs, now renamed xanthine riboswitches, function as genetic “OFF” switches to repress the translation of mRNAs whose gene products would otherwise generate oxidized purines. These findings reveal that some bacterial cells monitor the levels of oxidized purines, presumably to avoid accumulating unnecessary or even toxic levels of these purine degradation waste products.

## RESULTS AND DISCUSSION

### Genes associated with the *NMT1* motif share similarity with dioxygenases involved in purine oxidation

The consensus sequence and secondary structure model originally reported for *NMT1* motif RNAs (Weinberg et al. 2017a) was updated after the identification of additional unique-sequence representatives. These additional RNAs were uncovered and subsequently examined by using comparative sequence analysis algorithms to search recently released bacterial genomic sequence data (see Materials and Methods for details). A total of 649 representatives have now been identified, which are widely distributed in alpha-, beta- and gamma-Proteobacteria.

**FIGURE 1.**
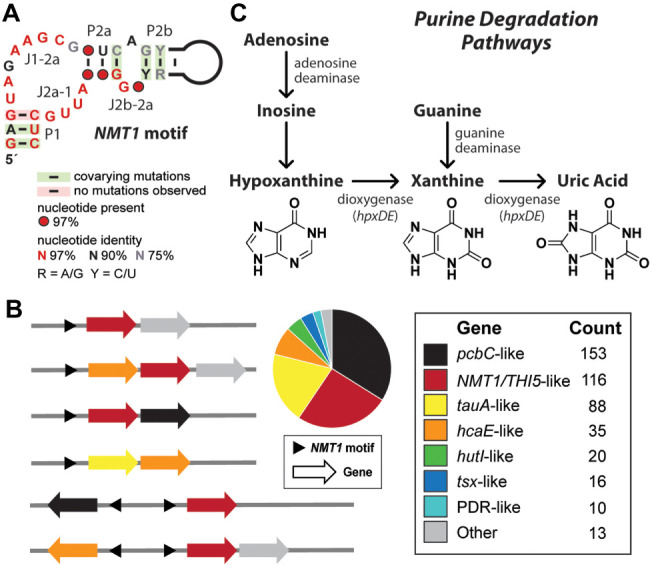
Characteristics of *NMT1* motif RNAs. (*A*) Updated consensus sequence and secondary structure model for the *NMT1* motif based on 649 unique representatives. (*B*) Downstream genetic contexts of *NMT1* motif representatives. (*Left*) Representative genetic arrangements reflect diverse gene associations, including operons. (*Right*) The pie chart depicts the abundances of the gene located immediately downstream from *NMT1* motif representatives. (*C*) Basic purine degradation and utilization pathways (Riva et al. 2008). Genes *hpxD* and *hpxE* code for known aromatic ring hydrolases.

The additional *NMT1* motif representatives were examined to create an updated consensus model and to expand the available information regarding gene associations. The revised secondary structure model roughly conforms to a two-stem (P1 and P2) junction, wherein stem P2 forms a small internal bulge to divide the stem into two parts (P2a and P2b) (Fig. 1A). Key differences between the new consensus model and the previous model are supported by nucleotide covariation that is consistent with the formation of P2a, and by the absence of covariation evidence that led to the reduction in proposed length for the P1 stem. Most of the highly conserved nucleotides in the updated consensus model are located in the bulged nucleotides forming the junction between P1 and P2a (J1-2a), and likewise in the junctions J2b-2a and J2a-1. We speculated that these highly conserved nucleotides are likely to be critical for the formation of the ligand-binding aptamer structure.

Bioinformatic comparisons between representatives also yielded evidence for the common use of expression platforms that use sequestration of the ribosome binding site (RBS) of the adjacent open reading frame (ORF) (Supplemental Fig. S1). This type of translation control mechanism is frequently used by members of many riboswitch classes (Barrick and Breaker 2007; Breaker 2018). Almost no examples of intrinsic terminator stems were identified, which is consistent with previous observations (Weinberg et al. 2017a) and with the fact that *NMT1* motif RNAs are found exclusively in Proteobacteria. These species rarely utilize expression platforms with a direct transcription control mechanism (Barrick and Breaker 2007).

As described previously (Weinberg et al. 2017a), genes located immediately downstream from *NMT1* motif representatives primarily belong to three gene types, which code for proteins with similarities either to isopenicillin N synthase and related dioxygenases (*pcbC*), to NMT1/THI5*-*like proteins (see above), or to ABC-type nitrate/sulfonate/bicarbonate transporters (*tauA*). Additional associated genes code for proteins similar to various dioxygenases (*hcaE*, PDR, DIOX_N), metallodependent amidohydrolases (*hutI*), amidohydrolases similar to 8-hydroxyguanine and guanine deaminases (PRK12393*)*, nucleoside transporters (*tsx*), and in rare instances tRNA adenosine deaminases (*tadA*). Notably, these associated genes sometimes are found in series following an *NMT1* motif RNA, indicating that some members of this candidate riboswitch class regulate an operon of genes related to the same metabolic process (Fig. 1B).

We further examined the genetic context of *NMT1* motif RNAs by using NCBI Protein Blast on the associated genes that were annotated as dioxygenases. These dioxygenases were previously predicted to be Rieske non-heme iron aromatic-ring-hydroxylating enzymes or alpha-ketoglutarate dependent dioxygenases that share sequence similarity with the *hpxD* and *hpxE* genes cluster discovered in *Klebsiella pneumoniae* (Riva et al. 2008). In *K. pneumoniae*, the *hpxDE* genes are involved in the oxidation of hypoxanthine to produce xanthine, and the oxidation of xanthine to yield uric acid (Fig. 1C; Riva et al. 2008). This additional information led us to pursue a hypothesis wherein the *NMT1* motif architecture represents a highly conserved aptamer domain that selectively binds an oxidation product resulting from purine nucleotide degradation.

### *NMT1* motif RNAs function as selective aptamers for xanthine and uric acid

**FIGURE 2.**
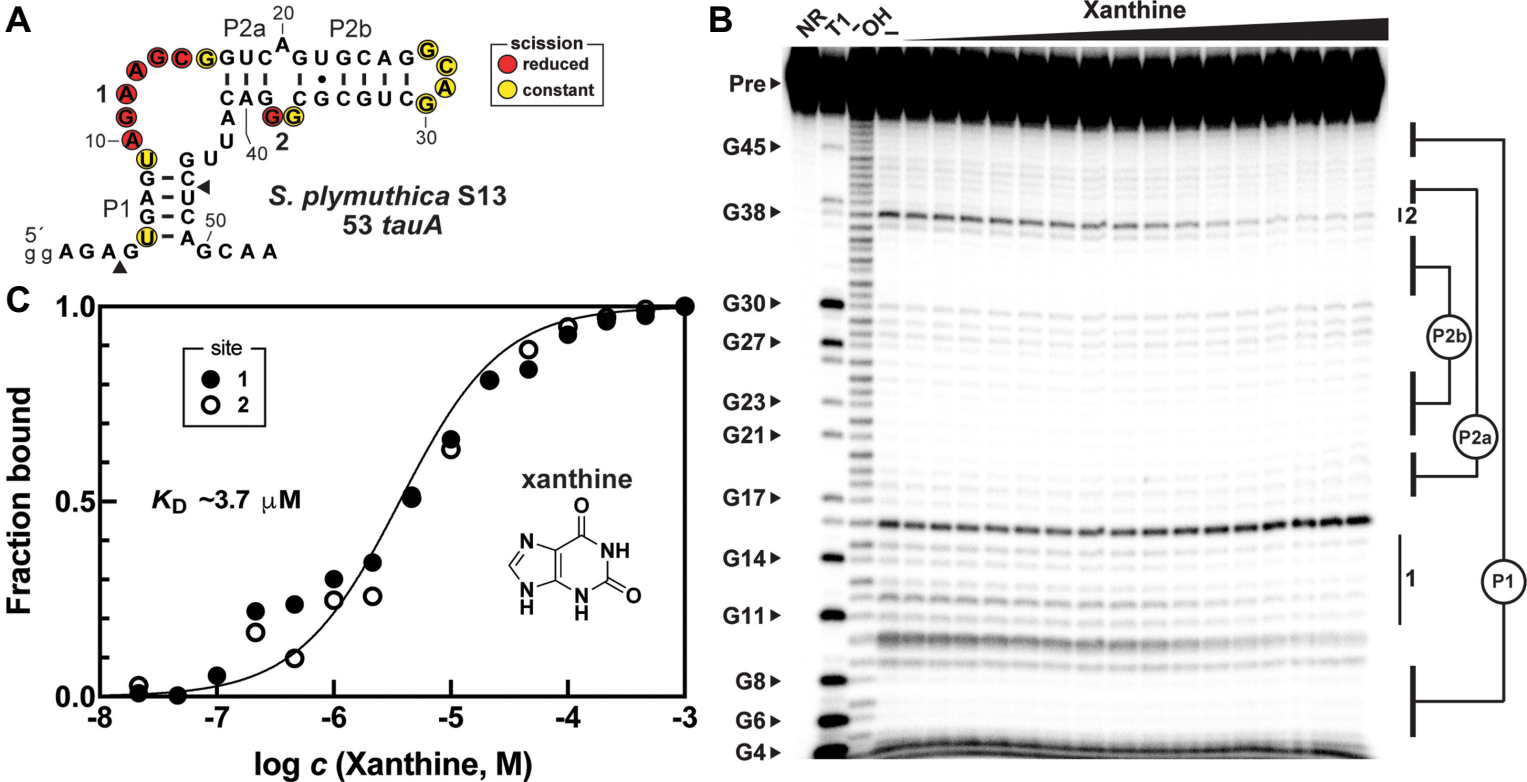
An *NMT1* motif RNA binds xanthine. (*A*) Sequence and secondary structure model of the 53 *tauA NMT1* motif RNA construct based on a representative from *S. plymuthica* S13, which is naturally located upstream of the *tauA* coding region. The 5′ terminus includes two guanosine nucleotides (lowercase letters) to improve in vitro transcription efficiency. Nucleotides whose phosphodiester linkages undergo scission during in-line probing reactions (see the PAGE image in *B*) are encircled and undergo modulation as indicated. (*B*) PAGE autoradiogram of 5′ ^32^P-labeled 53 *tauA* RNAs subjected to in-line probing reactions without (–), or with a range (10 nM to 1 mM) of xanthine concentrations. Lanes NR, T1, and ^−^OH indicate RNAs subjected to no reaction, limited digestion with RNase T1 (cleaves after each G), and incomplete digestion under alkaline conditions (cleaves after every nucleotide), respectively. The RNA precursor (Pre) band and select bands generated via RNase T1 digestion are indicated. Regions 1 and 2 correspond to sites of the RNA that exhibit structural modulation upon addition of xanthine. (*C*) Plot of the fraction of RNA bound to ligand versus the logarithm of the concentration of xanthine, as determined based on quantification of band intensity changes at sites 1 and 2 of the single experiment depicted in *B*. A trendline with a *K*_D_ of 3.7 µM (*R*^2^ = 0.9515) generated from a four-parameter logistic fit with the following parameters (minimum value equal to 0, maximum value equal to 1 and Hill coefficient equal to 1) is superimposed on the data points.

An RNA construct called 53 *tauA* (Fig. 2A) carrying 53 nt of the *NMT1* motif representative associated with the *tauA* gene from *Serratia plymuthica* strain S13 was prepared by in vitro transcription. 5′ ^32^P-labeled RNAs were then evaluated by using in-line probing, which is a method that provides information on the structural characteristics of RNA, including folding changes brought about by ligand binding (Soukup and Breaker 1999, Regulski and Breaker 2008). Initial examination of a diverse collection of purines revealed that modulation of the 53 *tauA* RNA structure is triggered by xanthine binding (Fig. 2B), which exhibits an apparent dissociation constant (*K*_D_) of ∼3.7 µM (e.g., Fig. 2C) based on two repeat experiments. Similar results were obtained for uric acid (*K*_D_ ∼ 25 µM), which differs from xanthine by oxidation of the C8 position of the purine ring (Supplemental Fig. S2).

**FIGURE 3.**
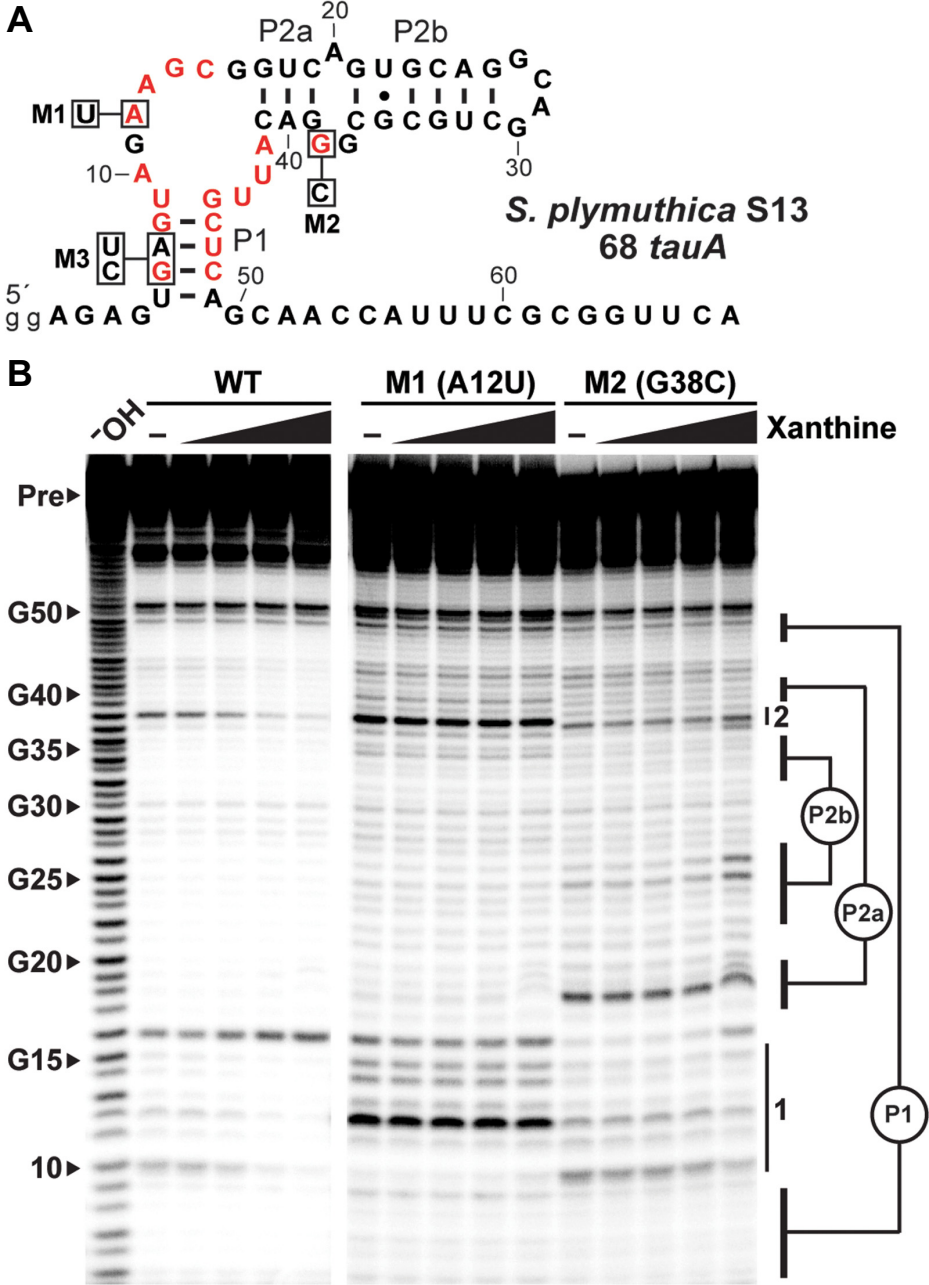
Mutations in the conserved core of an NMT1 motif RNA disrupts ligand binding. (*A*) Sequence and secondary model of the 68 *tauA* construct with the location of mutations in constructs M1 and M2. Boxed nucleotides identify the mutation relative to the highly conserved WT nucleotides (red). (*B*) In-line probing data derived from subjecting 5′ ^32^P-labeled 68 *tauA* RNA to incubations in the absence (–) or presence of xanthine as indicated. Arrowheads identify bands corresponding to the 5′ RNA fragments produced by strand scission at the nucleotide positions as indicated. Other annotations are as described in the legend to Figure 2B.

Ligand binding by the aptamer is dependent on the presence of highly conserved nucleotides in the joining regions. This assessment was made by examining mutant versions of an extended aptamer construct carrying 68 nt upstream of the *tauA* ORF from *S. plymuthica* S13 called 68 *tauA* (Fig. 3A). This extended construct was originally prepared and tested concurrently with the 53 *tauA* construct to reduce the experimental risk of testing only a single construct that might be trimmed too short or that might misfold. Mutations were arbitrarily tested using the longer of the two constructs.

Mutant construct M1 of the 68 *tauA* construct, which carries a single A to U mutation at position 12 that alters the nucleotide identity at an otherwise strictly conserved site, exhibits a complete loss of xanthine binding (Fig. 3B). Construct M2, which carries a G to C mutation at the invariant nucleotide position 38, likewise loses ligand binding function. Although there appears to be some modulation of the M2 construct in response to xanthine in the final lane (Fig. 3B), there are two reasons why we believe this does not reflect the normal function of the RNA. First, the banding pattern suggests that the structure adopted by the M2 construct is distinct from WT, and therefore the mutant construct is not expected to function normally. Second, the banding pattern changes are different than those for the unmodified construct. Given that this last lane has a very high concentration of xanthine, we believe the changes are indicative of artifacts in banding patterns typically observed when high ligand concentrations nonspecifically affect in-line probing reactions.

Similar losses in ligand binding function were observed for constructs M1 and M2 when exposed to uric acid (Supplemental Fig. S3). Likewise, construct M3 (Fig. 3A), in which the stability of P1 is disrupted, also loses xanthine binding function (Supplemental Fig. S4). These results are consistent with our hypothesis that the highly conserved secondary structure and core of *NMT1* motif RNAs forms a binding pocket for certain oxidized purines such as xanthine and uric acid.

**FIGURE 4.**
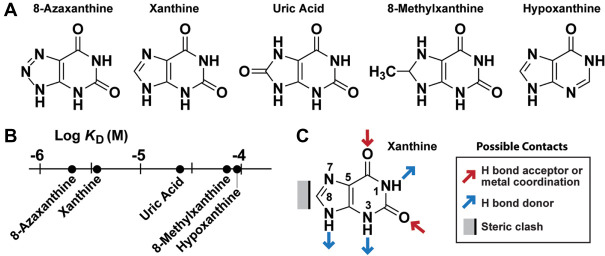
SAR data for ligand binding by an *NMT1* motif RNA representative. (*A*) Chemical structures xanthine and various analogs that are bound by the 53 *tauA* RNA construct. (*B*) Plot of the *K*_D_ values for various ligands. Compounds that failed to exhibit evidence of binding when tested at 1 mM are depicted in Supplemental Figure S4, Supplemental Figure S6, and Supplemental Figure S7. (*C*) Proposed molecular recognition contacts based on the affinities observed for various ligand candidates tested.

To further examine the ligand binding characteristics of *NMT1* motif RNAs, we conducted an SAR analysis by subjecting either the 53 *tauA* (Fig. 2A) or the 68 *tauA* (Fig. 3A) RNA constructs to in-line probing analyses in the presence of various analogs of xanthine or other nucleobases and their derivatives. Each compound was initially tested at concentrations of 100 µM and 1 mM to screen for ligand binding (e.g. see Supplemental Fig. S5), and additional active compounds (Fig. 4A) were further analyzed to establish *K*_D_ values (Fig. 4B; Supplemental Fig. S6). Most analogs tested, including the common natural pyrimidine nucleobases, are strongly rejected by the aptamers (Supplemental Figs. S5, S7), indicating that the ligand-binding pocket is highly selective for specific oxidized purine compounds.

The combined ligand binding data were then used to create a model depicting the proposed key molecular recognition contacts on xanthine (Fig. 4C). Notably, analogs of xanthine or uric acid that carry a single methyl group on the N1, N3, or N7 positions fail to associate with the aptamer (Supplemental Figs. S7, S8). Binding is also precluded by the presence of a methyl group on N9 of uric acid, or the presence of a ribose moiety at this same position on hypoxanthine (as the compound inosine). These results highlight the importance of these purine ring positions as molecular recognition contact points, although the additional bulk presented by methyl or ribose group appendages could cause a steric clash with the aptamer. Alteration of the carbonyl oxygen atoms on C2 and C6 is also disruptive, suggesting these oxygens might be involved in forming hydrogen bonds, or possibly forming contacts with Mg^2+^ ions as is common with structured RNAs such as riboswitches (Ferré-D'Amaré and Winkler 2011).

We also examined the ligand function of 8-azaxanthine, which is a small molecule inhibitor of uric acid oxidase, an enzyme that breaks down uric acid to 5-hydroxyisourate (Colloc'h et al. 1997; Ramazzina et al. 2006). This compound is bound with an affinity similar to xanthine and uric acid, whereas some compounds with added bulk at position 8 of the purine ring exhibit some loss of binding affinity (Fig. 4A,B). These results suggest that the C8 position alone is of little importance for molecular recognition, although changes at this position might have some effect on the ability of the adjacent atoms to contact the aptamer. Also, there might be a modest steric effect that permits the aptamer to discriminate against ligands with certain chemical moieties attached to C8. Regardless, the current SAR data suggests that the aptamer exploits various contacts with xanthine and closely related analogs to form a highly selective binding pocket for natural purine oxidation products (Fig. 4C).

In the process of validating *NMT1* motif RNAs as natural aptamers for xanthine and uric acid, we also tested other compounds chosen based on the genetic context, including those most relevant to the thiamin biosynthesis and histidine degradation pathways. However, we did not observe modulation of the 53 *tauA* RNA construct by these ligands at concentrations as high as 1 mM (Supplemental Fig. S5). These findings again support our conclusion that *NMT1* motif representatives function as high-affinity aptamers for xanthine and uric acid.

### Riboswitch-reporter fusion assays demonstrate that *NMT1* motif RNAs function as genetic “OFF” switches

**FIGURE 5.**
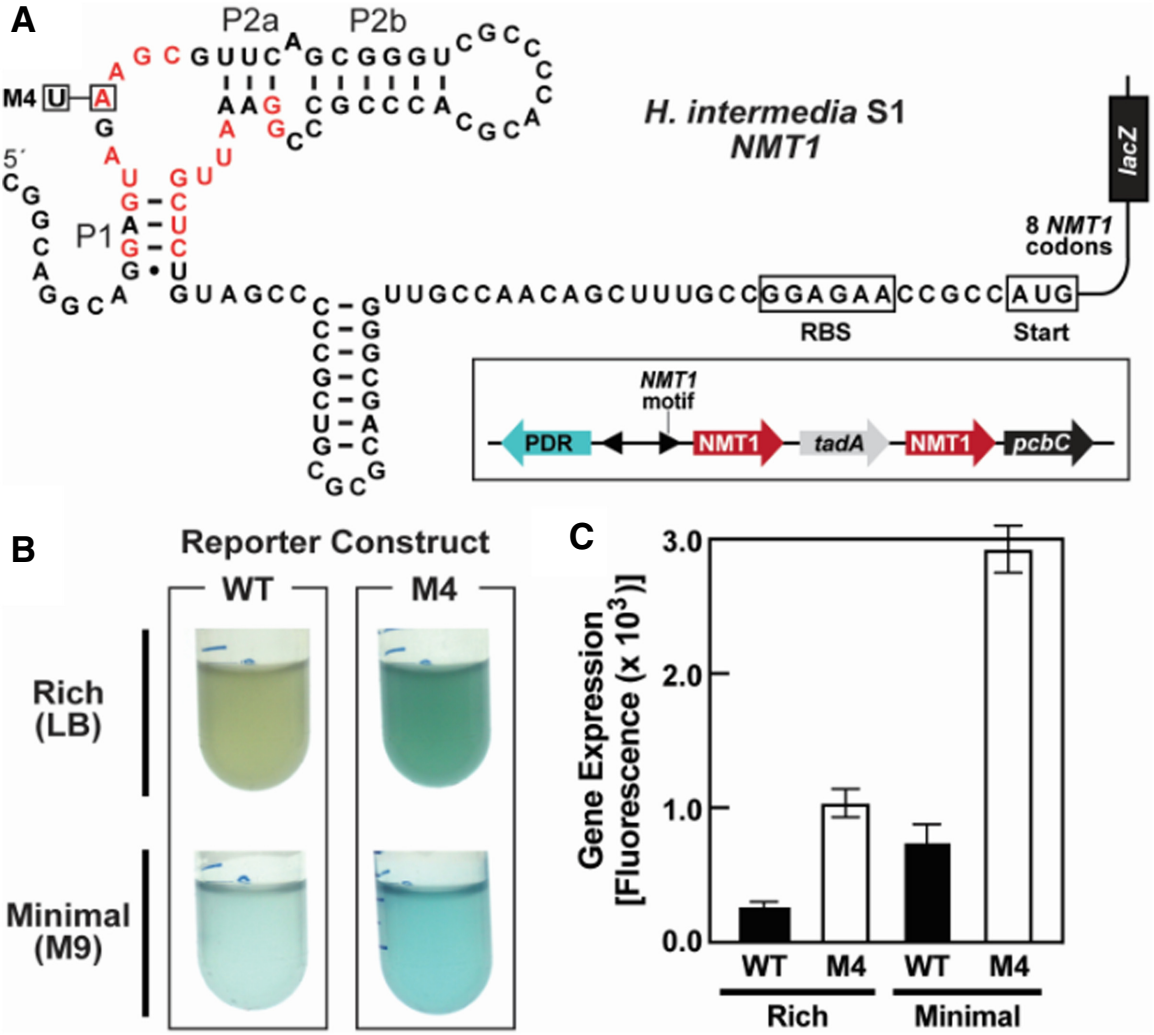
Reporter gene assays indicate that NMT1 motif RNAs are genetic “OFF” riboswitches. (*A*) Riboswitch-reporter fusion construct formed by linking the *NMT1* motif RNA representative from *H. intermedia* S1 to *lacZ* gene. Red letters identify the highly conserved nucleotides from the NMT1 motif consensus model (Fig. 1A). (*Inset*) The bacterium *H. intermedia* S1 carries adjacent NMT1 motif representatives in opposing orientations. The *right-most* representative was used for the reporter construct. (*B*) Liquid media assays of *E. coli* cells harboring either the wild-type (WT) or the a single conserved-nucleotide mutant (M1) reporter construct. Cells were grown in rich (LB) or minimal (M9) media conditions in the presence of the β-galactosidase activity indicator x-gal. (*C*) Plot of reporter gene expression for the WT and M4 constructs of the *H. intermedia* S1 riboswitch. Error bars represent the standard deviation for the measurements based on six replicate experiments.

To evaluate the possible genetic control functions of *NMT1* motif RNAs, we created a construct for gene expression studies in *Escherichia coli* as a surrogate organism by fusing the *NMT1* motif representative from *Hydrogenophaga intermedia* strain S1 to a β-galactosidase reporter gene (Fig. 5A). This *NMT1* motif sequence is naturally associated with four genes that are commonly found with *NMT1* motif representatives: two *NMT1* genes, *pcbC* and *tadA* (Fig. 5A, inset). Specifically, the *NMT1* and *pcbC* genes are relevant to purine oxidation as described above. The associated *tadA* gene is predicted to function as an adenosine deaminase, whose catalytic activity is expected to yield the oxidized nucleoside inosine. Therefore, the *NMT1* motif example chosen for genetic analysis a typical representative of this candidate riboswitch class.

The *H. intermedia* representative also was chosen in part because of the relatively short distance between the aptamer and the RBS. Specifically, a ribosome binding site (RBS) resides downstream from the conserved portion of the *NMT1* motif, suggesting that the expression platform might involve the regulation of ribosome binding to the mRNA. Furthermore, there is no evidence for the presence of an intrinsic terminator stem (strong stem followed by a run of U nucleotides) (Wilson and von Hippel 1995; Yarnell and Roberts 1999) in this or other examples (Supplemental Fig. S1), which suggests that members of this riboswitch class do not directly use a transcription termination mechanism.

Two genetic reporter constructs were tested: a wild-type (WT) version and a mutant version (M4) that carries a single nucleotide change at the equivalent location to the 53 *tauA* M1 construct, which is known to cause a loss of ligand binding activity (Fig. 3A). In liquid media assays, levels of the WT riboswitch reporter gene expression were lowest under both rich media and minimal media growth conditions. In contrast, the M4 reporter construct exhibits higher gene expression under both rich and minimal media conditions, suggesting that the riboswitch ligand is present under both conditions, and that the RNA motif turns “OFF” translation of downstream genes (Fig. 5B).

All the genes regulated by the *NMT1* motif are predicted to catalyze reactions on compounds that are earlier than xanthine and uric acid in the metabolic pathways for purine degradation. Therefore, the reporter assay results are consistent with the genetic contexts of *NMT1* motif RNAs. An excess of oxidized purine degradation products might need to trigger the repression of genes involved in the transport of nucleosides by nucleoside transporters, the deamination of purines by deaminases and amidohydrolases, and the oxidation of purines by dioxygenases, or the cell risks the toxic effects of these molecules. For example, uric acid is famously disruptive to biological systems due to its insolubility, and can cause joint and kidney damage in humans (Dalbeth et al. 2016).

### Concluding remarks

Taken together, our findings support the hypothesis that *NMT1* motif RNAs are selective riboswitches for oxidized purines such as xanthine and uric acid, and that they turn off genes whose expression would otherwise lead to the accumulation of potentially toxic levels of these compounds. However, it is notable that we do not observe genes related to oxidized purine degradation among the list of genes associated with this riboswitch class. We speculate that some species would have used representatives of this riboswitch class to activate genes involved in oxidized purine degradation or export if toxicity were the primary concern. This observation therefore suggests that cells might use this riboswitch class to monitor the production of xanthine, and possibly also uric acid, to maintain sufficient amounts for specific metabolic applications that are currently not readily apparent.

Given the ligand-binding specificity of these RNAs, we propose renaming *NMT1* motif RNAs as xanthine riboswitches. The discovery and experimental validation of xanthine riboswitches highlights some of the challenges encountered by those pursuing riboswitch ligand discovery and validation studies. In particular, vague or inaccurate gene annotations can cause confusion and delays with experimental efforts. In this case, the *NMT1/THI5*-like gene annotations were most misleading, as the xanthine riboswitch class appears to be unrelated to thiamin metabolism. However, careful comparisons made between the genes associated with this riboswitch class and other proteins with similar amino acid sequences provided clues that were sufficient to create a more promising collection of ligand candidates that could be tested.

Prior research had uncovered gene clusters in *K. pneumoniae* and *K. oxytoca* involved in hypoxanthine assimilation (Riva et al. 2008) and purine utilization (Pope et al. 2009), respectively. Although dioxygenases associated with *NMT1* motif representatives share sequence similarity with alpha-ketogluatarate dependent dioxygenases, the precise functions of these proteins have not been established. The finding that *NMT1* motif RNAs bind to xanthine and uric acid strongly supports the view that the dioxygenases will eventually be proven to operate on purine substrates. This hypothesis also is consistent with the fact that the dioxygenases associated with this riboswitch class are highly similar in sequence to dioxygenases encoded by *hpxD* and *hpxE* genes, which are known to be associated with purine oxidation pathways (Riva et al. 2008).

The strong binding affinities of *NMT1* motif RNAs to 8-azaxanthine, xanthine, and uric acid support the notion that the RNA binds to oxidized purines likely involved in the purine degradation pathway in these bacteria. However, we cannot be certain at this time whether members of this riboswitch have a specific preference in cells for the ligand that triggers gene control. It is possible that xanthine riboswitches respond to the accumulated pool of oxidized purines, or perhaps the natural ligand simply is the single oxidized purine form that accumulates the most. Regardless, our results will provide useful guidance as researchers perform further experiments to validate the functions of the proteins encoded by the riboswitch-controlled genes, which is warranted given the importance of purine metabolism in living systems.

## MATERIALS AND METHODS

### Bioinformatics analyses

Additional *NMT1* motif RNAs (Supplemental File S1) were discovered by using the comparative sequence analysis algorithms CMfinder (Yao et al. 2006) and Infernal 1.1 (Nawrocki and Eddy 2013) as described previously (Weinberg et al. 2017a). The database examined was comprised of a complete set of genomic DNA sequences (RefSeq version 80) and microbial environmental sequence collections as described previously (Weinberg et al. 2017a). RNA sequence and secondary structure consensus models and covariation data were depicted by using R2R software (Weinberg and Breaker 2011), and manually examined to assess the data and adjust the depictions. Protein sequence homology was determined using NCBI Basic Local Alignment Search Tool (BLAST) (Altschul et al. 1990).

### Chemicals, biochemicals, and oligonucleotides

Synthetic oligonucleotides (Supplemental Table S1) and most compounds used to conduct SAR analyses were obtained from Sigma-Aldrich. Exceptions include 3- and 9-methyluric acid (Santa Cruz Biotechnology, Inc.), isoxanthopterine and 7-methyluric acid (Cayman Chemical Company), and 8-hydroxyguanine (Carbosynth). [γ-^32^P] ATP (specific activity: 6000 Ci/mmol) was purchased from PerkinElmer. Genetic reporter constructs were designed (Supplemental Table S2) by the authors and subsequently purchased from GenScript VectorArt Gene Synthesis.

### RNA oligonucleotide preparation

Synthetic DNA oligonucleotides templates with a T7 RNA polymerase (T7 RNAP) promoter on the 5′ terminus were transcribed, purified by 10% polyacrylamide gel electrophoresis (PAGE), and extracted, using protocols similar to those previously described (Chen et al. 2019).

To generate 5′ ^32^P-labeled RNAs for in-line probing assays, 80 pmols of the RNA transcript was first dephosphorylated using rAPid alkaline phosphatase (Roche Life Sciences) following the manufacturer's protocol. Subsequently, 20 pmoles of the resulting RNA was ^32^P-radiolabeled at the 5′ terminus using T4 polynucleotide kinase in a 20 µL reaction mixture consisting of 25 mM CHES (pH 9.0 at 19°C), 5 mM MgCl_2_, 3 mM DTT, and 20 µCi [γ-^32^P]ATP. The reaction was incubated for 1 h at 37°C. The resulting radiolabeled RNA was purified by denaturing 10% PAGE and the desired RNA was extracted as noted above.

### In-line probing assays

In-line probing assays were performed as previously described (Soukup and Breaker 1999; Regulski and Breaker 2008) with the following exceptions. Stock solutions of ligand candidates were prepared in 50 mM aqueous sodium hydroxide. Reactions of 10 µL volume containing ∼125 nM of 5′ ^32^P-labeled RNA were incubated at 19°C for 36 h with the desired ligand in the presence of 100 mM Tris-HCl (pH 8.3 at 23°C), 100 mM KCl, and 20 mM MgCl_2_. Denaturing 10% PAGE was performed to resolve the reaction products. Binding curves and apparent *K*_D_ values were estimated as previously described (Malkowski et al. 2019) using ImageQuant 5.1 (GE Healthcare Life Sciences) for quantitation and GraphPad Prism 8 for graphical analysis.

### Genetic reporter assays

Genetic reporter constructs were designed with a *thiC* promoter from *E. coli*, followed by the *NMT1* motif RNA representative from *H. intermedia* strain S1, and included the first eight codons of the downstream ORF. This construct was inserted into 5′ EcoRI and 3′ BamHI restriction sites of plasmid pRS414 (Simons et al. 1987) upstream and in-frame with a *lacZ* (β galactosidase) gene by GenScript VectorArt Gene Synthesis. WT and M4 reporter constructs in plasmid pRS414 were transformed into *E. coli* strain BW25113 from the Keio Collection (Baba et al. 2006) and grown overnight in Luria Broth (LB) at 37°C with modest shaking. The following day, cultures were diluted to an OD_600_ of 0.1 and grown in both rich (LB) and minimal (M9) media in the presence of 50 µg mL^−1^ of X-gal (5-bromo-4-chloro-3-indolyl-β-d-galactopyranoside). Changes in culture color were recorded after 11 h incubations.

Liquid β-galactosidase assays were performed to quantify the observations in media supplemented with X-gal. Reporter gene expression was quantified as described previously (Nelson et al. 2015b; Perkins et al. 2019) with the following differences. Cells were grown overnight in rich media (LB) or minimal media (M9) for 18 h at 37°C while shaking at 220 rpm. 80 µL of a 1:100 diluted cell suspension (990 µL of fresh media with appropriate antibiotics plus 10 µL of the previous culture) was applied in triplicate to individual wells on a Costar black 96-well clear-bottom plate and incubated for 18 h. Following incubation, cell density and β*-*galactosidase activity measurements were performed as described elsewhere (Perkins et al. 2019).

## SUPPLEMENTAL MATERIAL

Supplemental material is available for this article.

## Supplementary Material

Supplemental Material
