# Supplementary material for: A bacterial riboswitch class senses xanthine and uric acid to regulate genes associated with purine oxidation
Source: RNA. 2020 Aug;26(8):960–8. doi: 10.1261/rna.075218.120 (PMC7373994; doi:10.1261/rna.075218.120)
Supplement: Supplemental Material [file supp_26_8_960__index.html]

A bacterial riboswitch class senses xanthine and uric acid to regulate genes associated with purine oxidation — Supplemental Material 

# A bacterial riboswitch class senses xanthine and uric acid to regulate genes associated with purine oxidation

## Supplemental Material

- Supplemental\_File\_S1.zip
- Supplemental\_Info\_and\_Tables.docx
- SupplementalFigS1.tif
- SupplementalFigS2.tif
- SupplementalFigS3.tif
- SupplementalFigS4.tif
- SupplementalFigS5.tif
- SupplementalFigS6.tif
- SupplementalFigS7.tif
- SupplementalFigS8.tif
