# Supplementary material for: A bacterial riboswitch class senses xanthine and uric acid to regulate genes associated with purine oxidation
Source: RNA. 2020 Aug;26(8):960–8. doi: 10.1261/rna.075218.120 (PMC7373994; doi:10.1261/rna.075218.120)
Supplement: Supplemental Material [file supp_075218.120_Supplemental_Info_and_Tables.docx]

**Supplementary Information**

**A bacterial riboswitch class senses xanthine and uric acid to regulate genes associated with purine oxidation**

**DIANE YU^1^ AND RONALD R. BREAKER^1,2,3^**

^1^Department of Molecular, Cellular and Developmental Biology, Yale University, New Haven, Connecticut 06520-8103, USA

^2^Department of Molecular Biophysics and Biochemistry, Yale University, New Haven, Connecticut 06520-8103, USA

^3^Howard Hughes Medical Institute, Yale University, New Haven, Connecticut 06520-8103, USA

**Corresponding author**: [ronald.breaker@yale.edu](about:blank)

**Supplemental Figure S1.** Examples of *NMT1* motif RNAs from three bacterial classes that are consistent with the use of an expression platform mechanism involving sequestration of the ribosome binding site (RBS) or the formation of an intrinsic terminator stem. Nucleotides shaded blue, orange and brown identify positions involved in the formation of P1, P2a and P2b, respectively. Some representatives exhibit short distances between the aptamer and the RBS and start codon features. In rare instances, the region between the conserved aptamer domain and the start codon for the adjoining ORF exhibits strong base-pairing followed by a run of U nucleotides (underlined). The lack of overlap of the aptamer and possible terminator stem structures is consistent with genetic “ON” riboswitch function.

**Supplemental Figure S2.** Uric acid binding by an *NMT1* motif RNA representative. (*A*) Sequence and secondary structure model of the 53 *tauA* RNA construct. (*B*) PAGE autoradiogram of 5ˊ ^32^P-labeled the 53 *tauA* RNAs subjected to in-line probing reactions without (–), or with a range (10 nM to 1 mM) of uric acid concentrations. (*C*) Plot of the fraction of RNA bound to ligand versus the logarithm of the concentration of uric acid. A trendline with a *K*_D_ of 4.3 μM (R-squared = 0.9366) generated from a four-parameter logistic fit with the following parameters (minimum value equal to 0, maximum value equal to 1 and Hill coefficient equal to 1) is superimposed on the data points. Other annotations and details are as described in the legend to **Fig. 2**.

**Supplemental Figure S3.** Mutations to conserved nucleobases in an *NMT1* motif RNA abolishes binding to uric acid. Depicted is an autoradiogram of PAGE-resolved in-line probing reaction products for the M1 and M2 versions of the *S. plymuthica* S13 *NMT1* 68 *tauA* RNA (**Fig. 3A**) incubated without (‒) and with 1, 10, 100 and 1000 μM of uric acid (left to right). Additional annotations are as described in the legend to **Fig. 3**.

**Supplemental Figure S4.** In-line probing analysis of the M3 version of the *S. plymuthica* S13 *NMT1* 68 *tauA* RNA (**Fig. 3A**) incubated without (‒) and with xanthine concentrations ranging from 10 nM to 1 mM (left to right). Additional annotations are as described in the legend to **Fig. 3**. Note that this RNA construct, which carries a disrupted P1 stem, fails to bind xanthine at concentrations as high as 1 mM.

**Supplemental Figure S5.** In-line probing assays to screen for binding of xanthine ligand analogs. (*A*) The 53 *tauA* RNA construct from *S. plymuthica* S13 used for in-line probing assays. Red nucleotides identify those positions that are highly conserved as depicted in **Fig. 1A**. Additional annotations are as described for **Fig. 2A**. (*B*) Compounds examined for ligand binding function in the experiment depicted in C. Note that all compounds were tested by in-line probing at a concentration of 1 mM unless otherwise indicated. (*C*) Autoradiogram of PAGE-resolved in-line probing reaction products for reactions incubated in the presence of 100 μM or 1 mM of the compounds as noted. In the data presented here, hypoxanthine, xanthine, uric acid, and 2-oxo-2,3-dihydro-1H-imidazole-4-carboxylic acid (compounds 11, 12, 13 and 15, respectively) exhibit evidence of binding. (*D*) Additional compounds tested that failed to exhibit evidence for ligand binding.

**Supplemental Figure S6 (previous page).** In-line probing assays were used to establish the *K*_D_ values for various ligands bound by the 53 *tauA* RNA construct. The ligands 8-azaxanthine (*A*), hypoxanthine (*B*), and 8-methylxanthine (*C*) were subjected to in-line probing assays and the bands quantitated to estimate the fraction of ligand bound are highlighted in A. For data in B and C that did not result in binding saturation, the overlaid trendline was generated from a four-parameter logistic fit with adjusted parameters where no maximum value was set. Other parameters remained identical to **Fig. 2** and **Supplemental** **Fig. S2**.

**Supplemental Figure S7.** In-line probing assays to screen for binding of xanthine ligand analogs. (*A*) The 53 *tauA* RNA construct from *S. plymuthica* S13 used for in-line probing assays. Additional annotations are as described for **Supplemental Fig. S4**.

**Supplemental Figure S8.** Chemical structures of compounds that failed to be bound by the *S. plymuthica* S13 *NMT1* *tauA* RNA (either 53 *tauA* or 68 *tauA* constructs) (**Fig. 2A**, **Fig. 3A**). Compounds were examined for binding by in-line probing using concentrations of 100 μM and 1 mM.

**Supplemental Table S1.** Sequences of synthetic DNA oligonucleotides used in this study. Bold letters designate the T7 RNA polymerase promoter and lowercase letters identify guanosine nucleotides added to facilitate *in vitro* transcription. Letters highlighted in yellow are mutated relative to the WT sequence.

| **Construct** | **Sequence (5′ to 3′)** |
| --- | --- |
| ***S. plymuthica* S13**  **53 *tauA*** | **TAATACGACTCACTATA**ggAGAGTGAGTAGAAGCGGTCAGTGCAGGCAGCTGCGCGGGACATTGCTCAGCAA |
| ***S. plymuthica* S13**  **68 *tauA*** | **TAATACGACTCACTATA**ggAGAGTGAGTAGAAGCGGTCAGTGCAGGCAGCTGCGCGGGACATTGCTCAGCAACCATTTCGCGGTTCA |
| ***S. plymuthica* S13**  **68 *tauA* A12U M1** | **TAATACGACTCACTATA**ggAGAGTGAGTAGTAGCGGTCAGTGCAGGCAGCTGCGCGGGACATTGCTCAGCAACCATTTCGCGGTTCA |
| ***S. plymuthica* S13**  **68 *tauA* G38C M2** | **TAATACGACTCACTATA**ggAGAGTGAGTAGAAGCGGTCAGTGCAGGCAGCTGCGCGCGACATTGCTCAGCAACCATTTCGCGGTTCA |
| ***S. plymuthica* S13**  **68 *tauA* G6C, A7U M3** | **TAATACGACTCACTATA**ggAGAGTCTGTAGAAGCGGTCAGTGCAGGCAGCTGCGCGGGACATTGCTCAGCAA |

**Supplemental Table S2.** Sequence of riboswitch-reporter gene constructs. 5ˊ EcoRI and 3ˊ BamHI restriction sites are in bold font. First eight codons of the downstream gene are shaded gray. The mutation is shaded yellow.

| **Construct** | **Sequence (5′ to 3′)** |
| --- | --- |
| ***H. intermedia* strain S1 WT**  **(*E. coli thiC* promoter)** | TACGAC**GAATTC**CAAAAATAATGCTGCTTGAGTTCTGCGCTG  TTAACGCGTAATTTACATTCAACGGCAGGAGTAGAAGCGTTC  AGCGGGTCGCCCCACGCACCCGCCCGGAAATTGCTCTGTAGC  CCCCGCTGCGCGCAGCGGGTTGCCAACAGCTTTGCCGGAGAA  CCGCCATGCAGCGCCGCCGCTTCCTCGCCGG**GGATCC**CCCGG  C |
| ***H. intermedia* strain S1 M3**  **(*E. coli thiC* promoter)** | TACGAC**GAATTC**CAAAAATAATGCTGCTTGAGTTCTGCGCTG  TTAACGCGTAATTTACATTCAACGGCAGGAGTAGTAGCGTTC  AGCGGGTCGCCCCACGCACCCGCCCGGAAATTGCTCTGTAGC  CCCCGCTGCGCGCAGCGGGTTGCCAACAGCTTTGCCGGAGAA  CCGCCATGCAGCGCCGCCGCTTCCTCGCCGG**GGATCC**CCCGG  C |
