# Supplementary figures and images for: A bacterial riboswitch class senses xanthine and uric acid to regulate genes associated with purine oxidation
Source: RNA. 2020 Aug;26(8):960–8. doi: 10.1261/rna.075218.120 (PMC7373994; doi:10.1261/rna.075218.120)

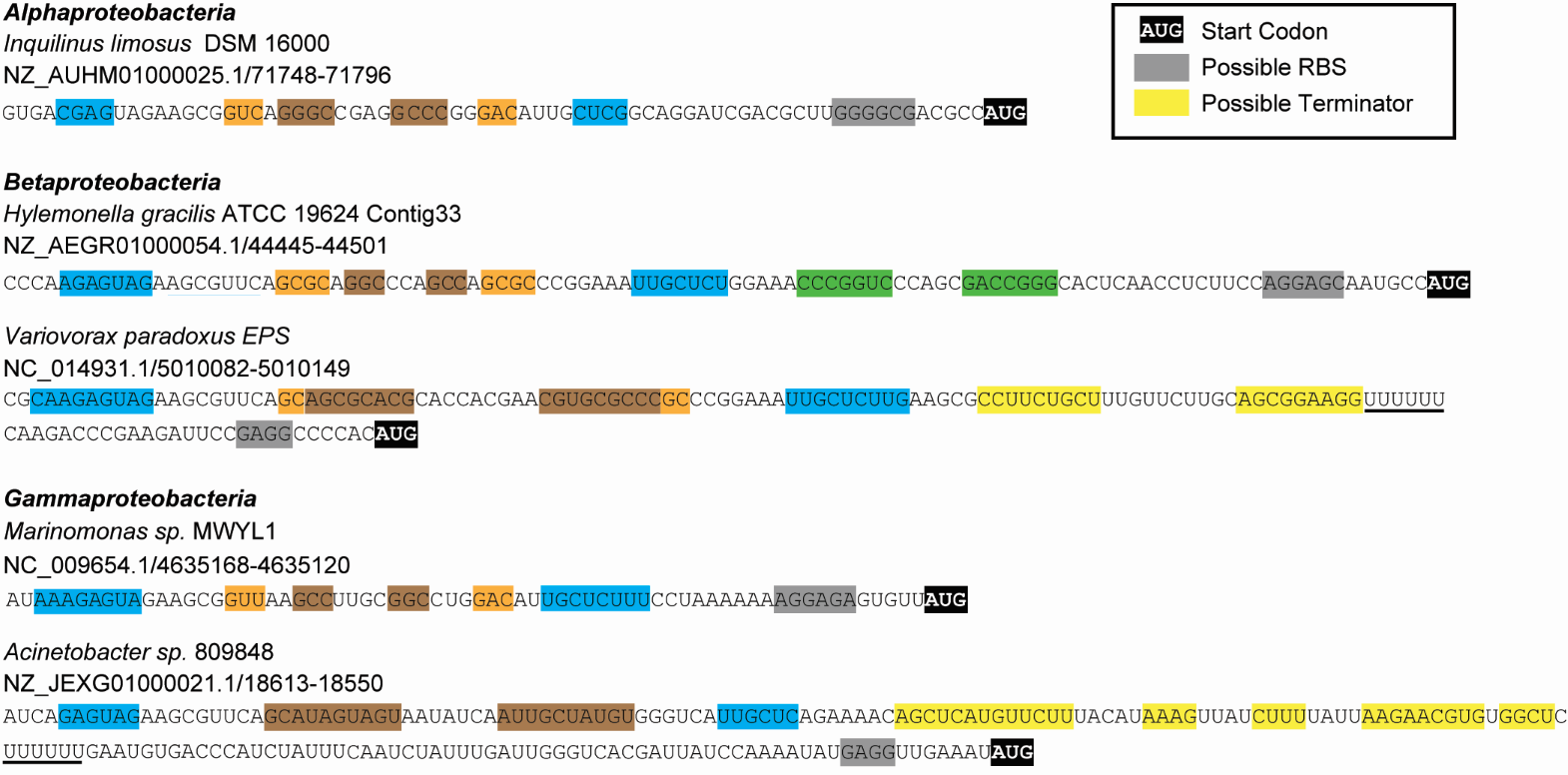

Supplement: Supplemental Material [file supp_075218.120_SupplementalFigS1.tif]

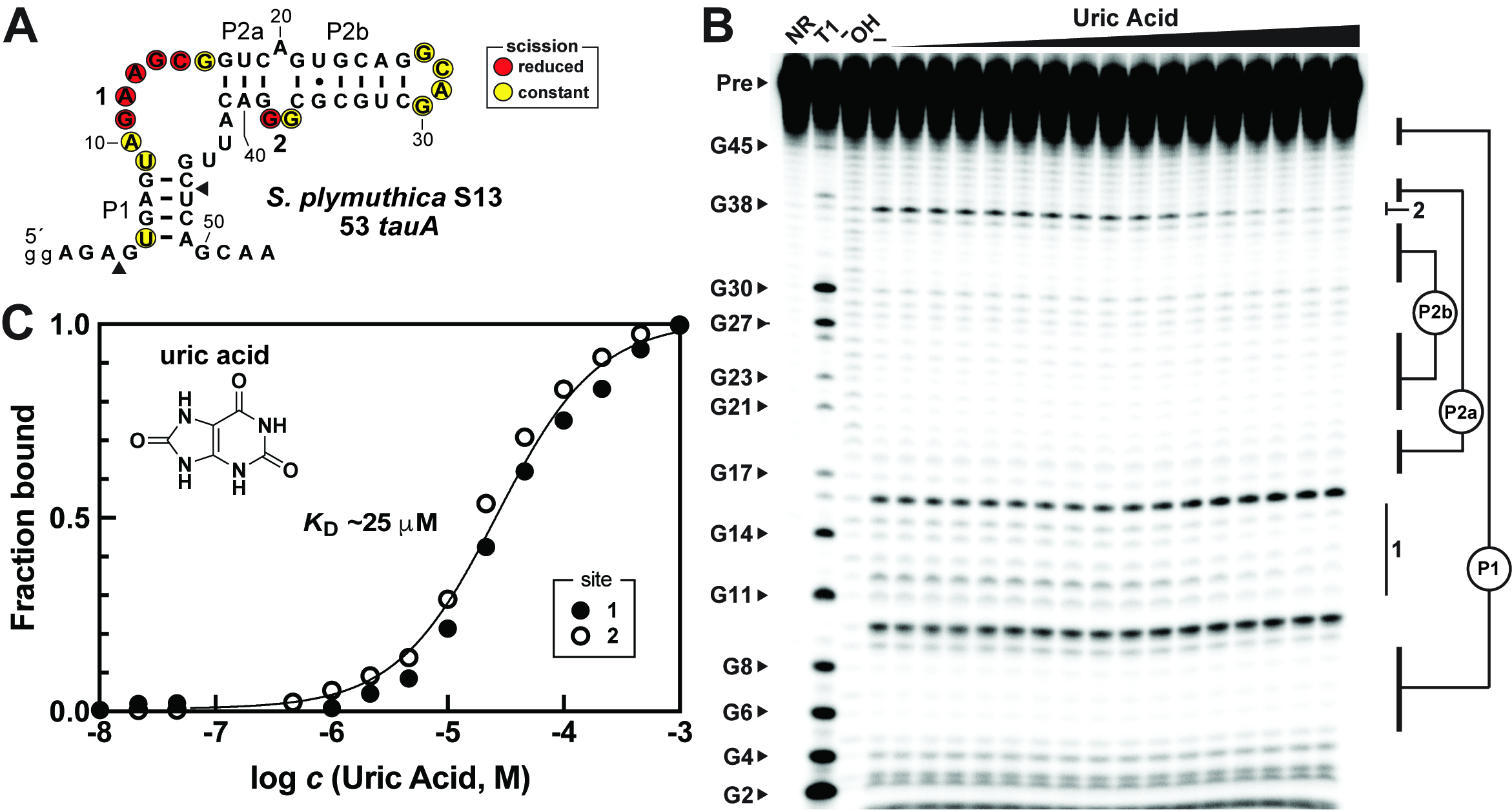

Supplement: Supplemental Material [file supp_075218.120_SupplementalFigS2.tif]

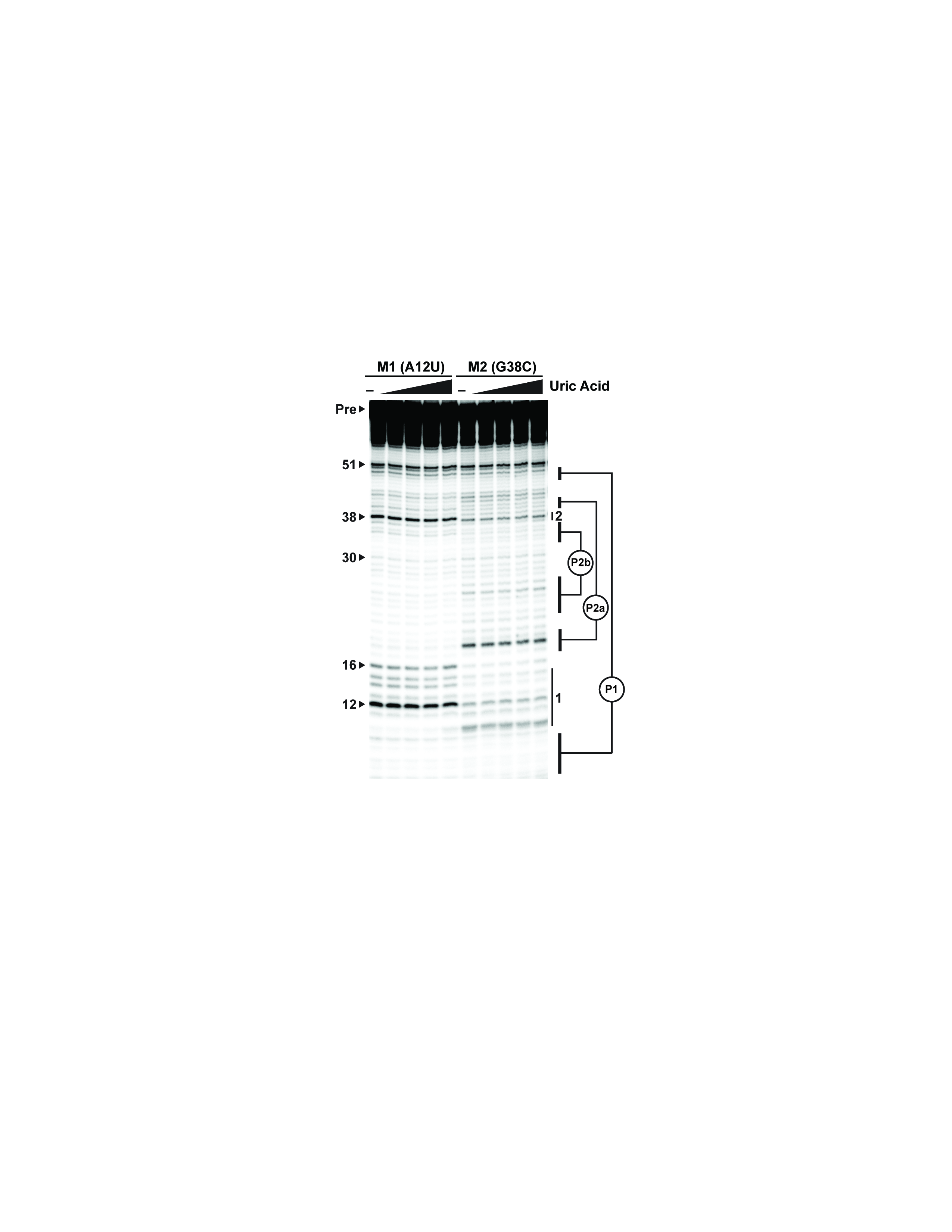

Supplement: Supplemental Material [file supp_075218.120_SupplementalFigS3.tif]

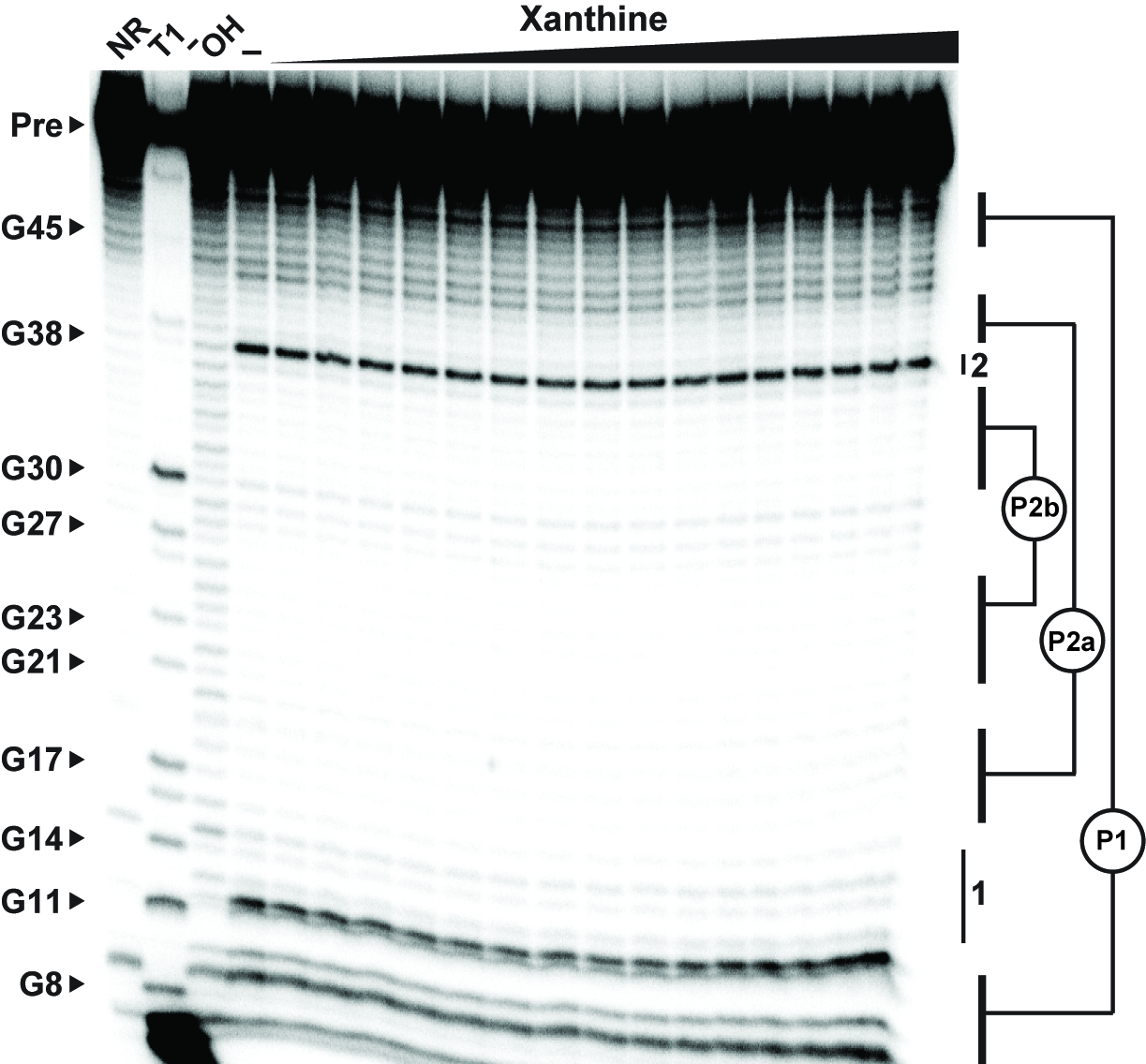

Supplement: Supplemental Material [file supp_075218.120_SupplementalFigS4.tif]

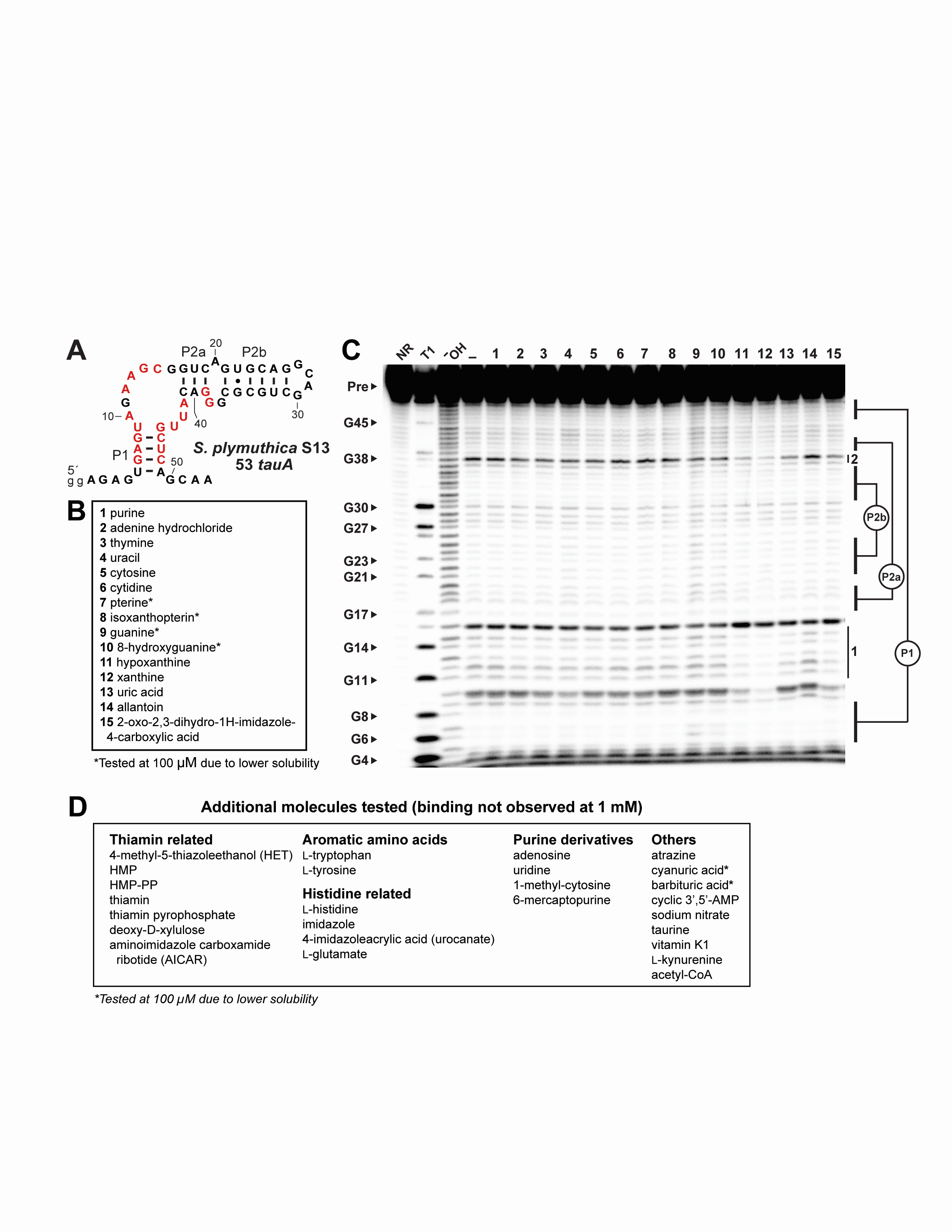

Supplement: Supplemental Material [file supp_075218.120_SupplementalFigS5.tif]

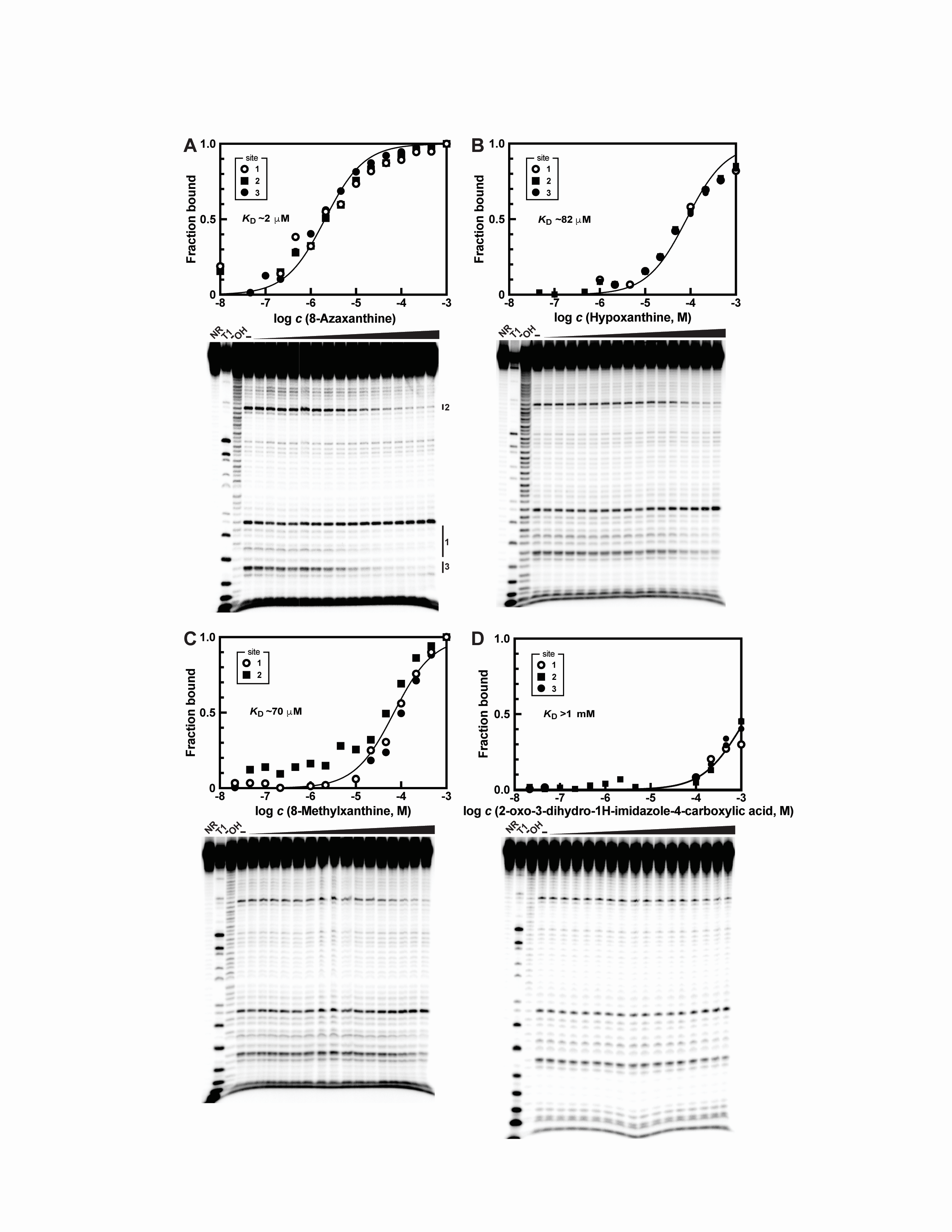

Supplement: Supplemental Material [file supp_075218.120_SupplementalFigS6.tif]

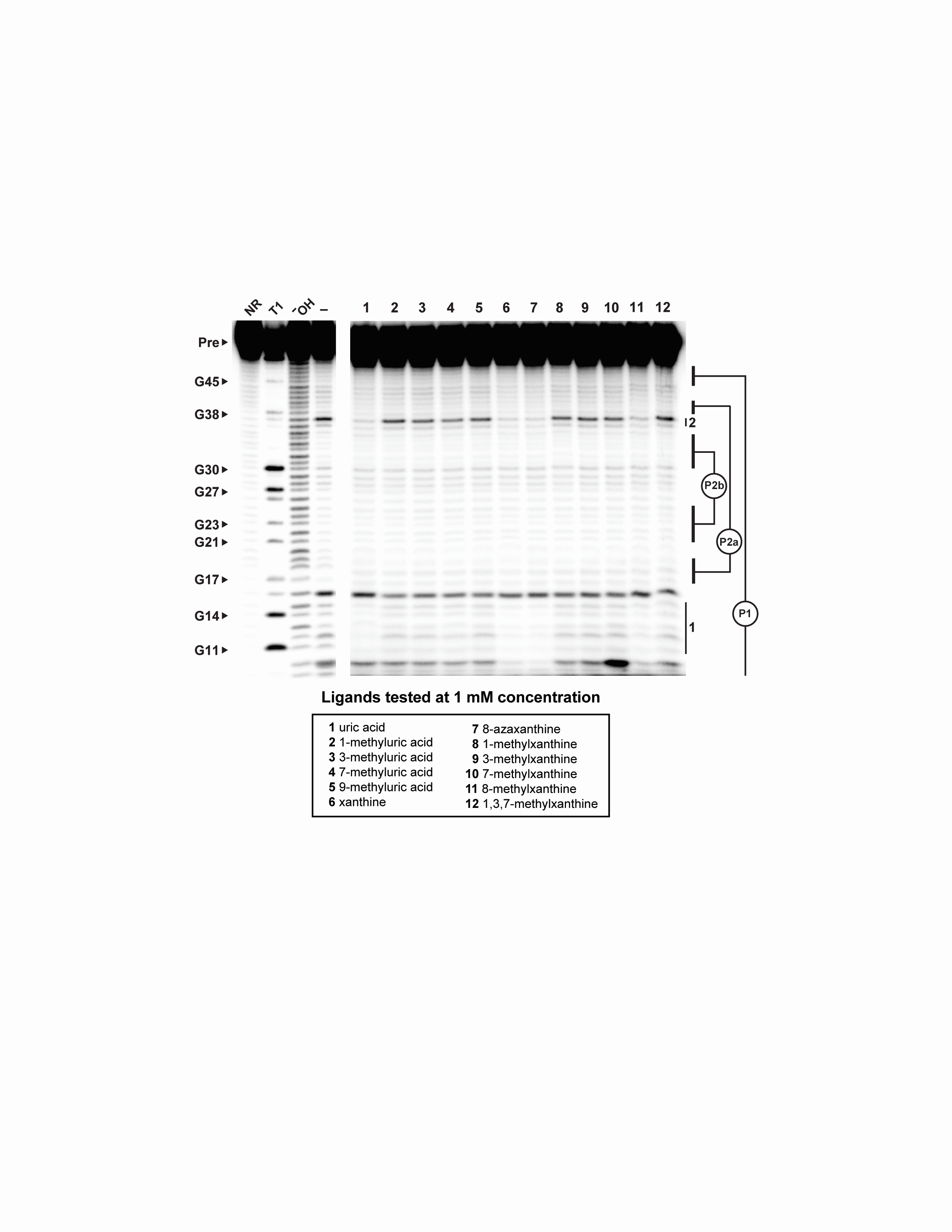

Supplement: Supplemental Material [file supp_075218.120_SupplementalFigS7.tif]

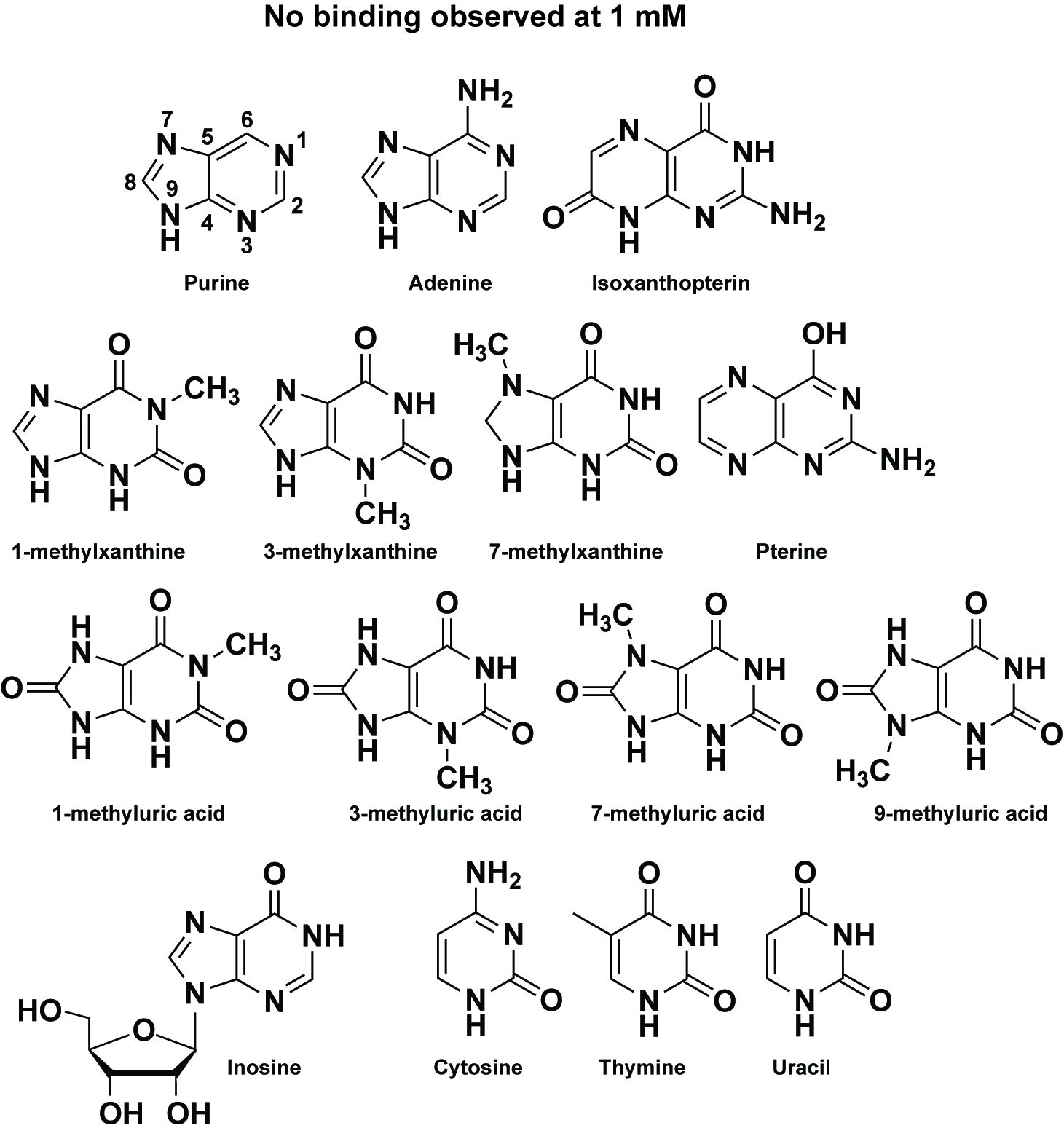

Supplement: Supplemental Material [file supp_075218.120_SupplementalFigS8.tif]
